# A generic method for assignment of reliability scores applied to solvent accessibility predictions

**DOI:** 10.1186/1472-6807-9-51

**Published:** 2009-07-31

**Authors:** Bent Petersen, Thomas Nordahl Petersen, Pernille Andersen, Morten Nielsen, Claus Lundegaard

**Affiliations:** 1 Center for Biological Sequence Analysis – CBS, Department of Systems Biology, Kemitorvet 208, Technical University of Denmark – DTU, DK-2800 Lyngby, Denmark; 2 Centre for Medical Parasitology – CMP, CSS Building 22, University of Copenhagen, DK-1014 Copenhagen, Denmark

## Abstract

**Background:**

Estimation of the reliability of specific real value predictions is nontrivial and the efficacy of this is often questionable. It is important to know if you can trust a given prediction and therefore the best methods associate a prediction with a reliability score or index. For discrete qualitative predictions, the reliability is conventionally estimated as the difference between output scores of selected classes. Such an approach is not feasible for methods that predict a biological feature as a single real value rather than a classification. As a solution to this challenge, we have implemented a method that predicts the relative surface accessibility of an amino acid and simultaneously predicts the reliability for each prediction, in the form of a Z-score.

**Results:**

An ensemble of artificial neural networks has been trained on a set of experimentally solved protein structures to predict the relative exposure of the amino acids. The method assigns a reliability score to each surface accessibility prediction as an inherent part of the training process. This is in contrast to the most commonly used procedures where reliabilities are obtained by post-processing the output.

**Conclusion:**

The performance of the neural networks was evaluated on a commonly used set of sequences known as the CB513 set. An overall Pearson's correlation coefficient of 0.72 was obtained, which is comparable to the performance of the currently best public available method, Real-SPINE. Both methods associate a reliability score with the individual predictions. However, our implementation of reliability scores in the form of a Z-score is shown to be the more informative measure for discriminating good predictions from bad ones in the entire range from completely buried to fully exposed amino acids. This is evident when comparing the Pearson's correlation coefficient for the upper 20% of predictions sorted according to reliability. For this subset, values of 0.79 and 0.74 are obtained using our and the compared method, respectively. This tendency is true for any selected subset.

## Background

For decades, machine learning has been used as a tool in bioinformatics for predictive purposes. A number of concepts have been implemented in order to estimate the predictive power of the individual methods. The commonly used performance measures have been described in Lundegaard et al. [1] Predictive power is generally estimated from a number of examples that have been excluded from the training process and an overall estimate of the accuracy of the method is calculated. This, however, will not provide information regarding the reliability of each of the individual predictions. For discrete qualitative predictions, the reliability is conventionally estimated as the difference between output scores of selected classes [2]. However, many biological problems are quantitative in nature and are therefore more appropriately characterized by a real value than a discrete class. Real value predictions often provide a single output value and the estimation of the accuracy of a given prediction is more complicated than for predictions of discrete classes. Prediction of the solvent accessible surface area (ASA) of amino acid residues within a native folded protein is an example of a real value prediction problem, where the estimation of reliability scores is nontrivial. The ASA for experimentally solved structures is given in Å^2 ^and the area is calculated by rolling a sphere the size of a water molecule over the protein surface [3]. For comparative and predictive purposes, the ASA is often transformed to a relative surface area (RSA), which is calculated as the ASA of a given amino acid residue in the polypeptide chain, relative to the maximal possible exposure of that residue in the center of a tri-peptide flanked with either glycine [4] or alanine [5]. Knowledge of the degree of surface exposure of an amino acid is valuable and it has been used to enhance the understanding of a variety of biological problems including protein-protein interactions [6,7], structural epitopes [8], active sites [9], and prediction of disease-related single nucleotide polymorphisms [10].

Several methods for predicting surface accessibility from the primary protein sequence have been developed often inspired by the related field of protein secondary structure prediction as exemplified with [11] implemented in [12]. Generally, the best methods involve the use of advanced machine learning algorithms such as artificial neural networks (ANN) or support vector machines (SVM) combined with evolutionary information [13-20]. The surface accessibility has traditionally been predicted in two classes as either buried or exposed using various more or less arbitrary cut-offs. Recently, real value RSA predictors have been developed thus removing the need to define specific cut-offs [5]. This change in focus from classifying towards quantitative systems has made it difficult to assess the reliability of a prediction. Previous studies have shown that prediction of the RSA is significantly more accurate for buried compared to exposed amino acids [21]. However, the most biologically interesting residues are often exposed, as these are able to interact with the environment. For this reason, it is important to have a good estimate of the reliability, especially for the more exposed amino acid residues. The current best method available for real value surface exposure prediction is Real-SPINE [22,23]. This method exists in a web accessible form, which in addition to the predicted surface accessibility, also provides a score for each prediction that is a measure of the consistency between two predictors (A, B). RS = 1 - |A - B| where A and B are the results from two predictors on solvent accessibility [22]. As described this score is solely a consistency score and it has not previously been described to what degree such consistency measures provide information of the reliability of the individual predictions beyond the fact that the most exposed residues are predicted most unreliably.

Here, we have developed a generic method that assigns a reliability score to each surface accessibility prediction as an inherent part of the training process. The method is evaluated on a common set of sequences and compared to other state-of-the art prediction methods. In particular, we investigate to what extent our method for residue-specific reliability prediction is able to discriminate between good and bad predictions in the entire range from completely buried to fully exposed amino acids.

## Results

**Figure 1:**
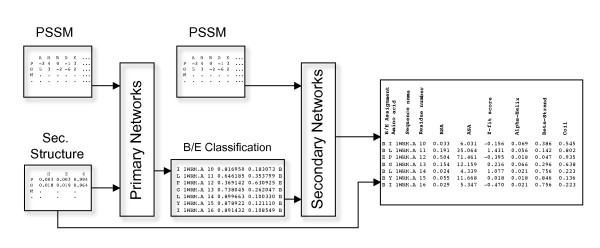
**Graphical overview of the method**. Graphic overview of the method used in training of the primary and secondary neural networks. 'PSSM' is a Position-Specific Scoring Matrix. 'Sec. Structure' is the raw output from secondary structure predictions. 'Primary Networks' are an ensemble of artificial neural networks (ANN) and 'B/E Classification' is the raw buried/exposed output from these ANNs. 'Secondary Networks' are also an ensemble of ANNs, trained to predict the relative surface exposure of an amino acid. The last box shows output from the web server.

A schematic overview of the NetSurfP method is shown in Figure 1. The method consists of two neural network ensembles. The primary networks are trained on sequence profiles and predicted secondary structure and have two outputs corresponding to buried or exposed, respectively. The higher output defines the predicted category. The secondary networks use these outputs as input together with sequence profiles and have been trained to predict the relative surface exposure of the individual amino acid residues. The proposed reliability prediction method is applied to the secondary networks only.

### Primary networks

Classification artificial neural networks (ANNs) were trained to predict whether an amino acid was buried or exposed i.e., below or above 25% of ASA_max _of the given amino acid. Input to the ANNs was sequence profiles and predicted secondary structures. The prediction performance of the secondary structure prediction in terms of the straight Q3 measure on the CB513 dataset was 81%. Secondary structure predictors were trained to predict H or E classes (see methods), which differs from the CASP classification scheme used by many secondary structure prediction methods (CASP Q3 = 78%).

Using 10-fold cross validation each spanning a series of different network architectures, an ensemble were constructed of the 200 best performing network architectures, determined by the cross validation leave-out test sets (see methods). A test performance of 79.8% accuracy and a Matthews correlation coefficient (MCC) of 0.593 were obtained. This ANN ensemble was also evaluated using the evaluation set CB513. The performance values were 79.0% correctly classified residues and a MCC of 0.577. These values are compared with the performance obtained by [22] as shown in table 1.

**Table 1:** Evaluated performance for the primary networks.

Method	% Correct	MCC
NetSurfP Classification CB513	79.0	0.577
Dor and Zhou [21]	78.8	-

Evaluation of the best performing ANN ensemble using the evaluation set CB513. The columns are the overall %-correct prediction of buried and exposed amino acids and Matthew's correlation coefficient (MCC). Dor and Zhou gives the performance value published by [22].

### Secondary networks

The output classification values from the primary networks were used together with sequence profiles in the form of Position-Specific Scoring Matrices (PSSM) to train the secondary neural networks as also implemented by [21]. A significant improvement was obtained compared to bare PSSM input only with respect to linear as well as two-state correlations (data not shown). Several neural network architectures were trained using 10-fold cross-validation. The best cross-validation leave out test set performance was obtained by using a window size of 11 residues and a number of hidden neurons in the range 25–200. The Real-SPINE method [22] has not previously been evaluated on the CB513 set. We therefore submitted the sequences in the CB513 set to the Real-SPINE 1.0 web-server.

Two sequences were not accepted by the server leaving us with a set of 511 sequences (CB511) used when comparing the performance of NetSurfP and several other methods [5,20,22,24]. The RealSpine and NetSurfP methods perform equally well as shown in table 2.

**Table 2:** Evaluation of NetSurfP and other surface accessibility predictors.

Method	Exposure	Train	CB513/CB511	Method
Ahmad	ASA	-	0.48	ANN
Yuan	ASA	-	0.52	SVR
Nguyan	ASA	-	0.66	Two-Stage SVR
Real-SPINE	ASA	0.74	0.73	ANN
Real-SPINE	RSA	-	0.70	ANN
NetSurfP	ASA	0.75	0.72	ANN
NetSurfP	RSA	0.72	0.70	ANN

Performances are shown for 5 different approaches to predict absolute and relative (RSA) surface accessibility. Methods included in the benchmark are Ahmad: [5], Yuan: [20], Nguyen: [24], Real-SPINE: [22], NetSurfP: This work. Train gives the training performance, and CB513/CB511 gives the evaluation performance on the CB513 data set. Train performance of the Real-SPINE method and evaluation performances for the Ahmad, Yuan, and Nguyen method are taken from the corresponding publications. ANN = Artificial neural networks, SVR = Support vector regression. Pearson's correlation coefficients (PCC) are shown for all methods based on the absolute surface exposure of an amino acid. Also, PCC values are given for relative surface exposure for the two methods NetSurfP and Real-SPINE.

### Prediction and analysis of reliability scores

Neural networks were trained as described in section 'secondary networks'. Real value predictions usually gives one output value between 0–1 per residue, however, our described method generates two output values for each prediction; the predicted surface accessibility and a reliability of this prediction for each amino acid residue. This was implemented using a modified back-propagation procedure as described in the method section. We evaluated the performance of this method on the CB511 data set and compared the results to those obtained with the method by Dor and Zhou [21]. Unless otherwise stated, the performance values were calculated from the RSA. The overall predictive performance of the neural network was 0.145 in terms of the mean error, E, and 0.70 in terms of the Pearson's correlation coefficient (PCC), which is similar to the values obtained earlier using the conventional networks (see table 2).

**Figure 2:**
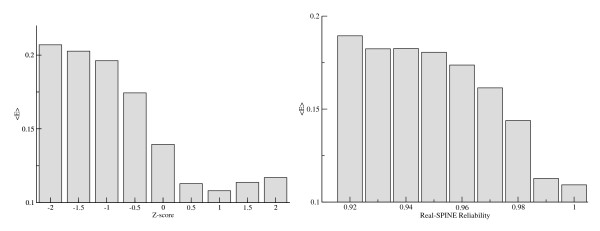
**The average error as a function of the predicted reliability**. The left panel shows NetSurfP Z-score versus mean error, and the right panel shows the consistency reliability score versus mean error.

From the network reliability score, we calculated a reliability value as a Z-score as described in methods. Figure 2 (left panel) shows the variation in the mean error as a function of the Z-score reliability from NetSurfP. From this figure, it is apparent that data points with high Z-scores have lower predicted error compared to data points with low Z-scores. We found that the group of data points with positive Z-scores, corresponding to 51% of all data points, achieved a PCC of 0.77, whereas the data points with negative Z-scores achieved a PCC of 0.64. This difference is highly significant (p < 0.001, Bootstrap exact estimate).

The Real-SPINE method provides a residue-specific consistency measure associated with each prediction. The relationship between this value and the mean error is shown in the right panel of Figure 2. Comparing these two plots suggests that both methods are able to identify the most reliable predictions.

**Figure 3:**
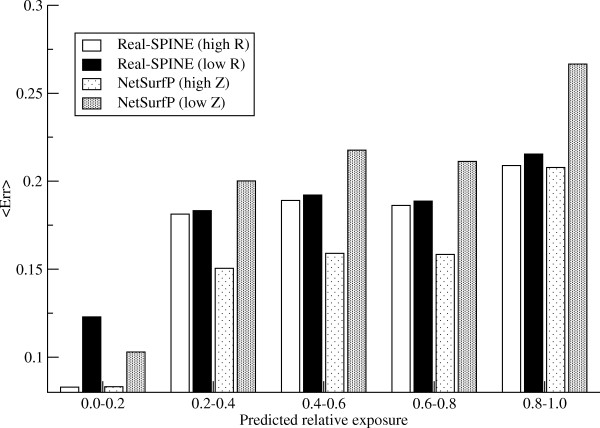
**Histogram of mean error as a function of predicted exposure values**. The bars show the histogram for four groups of predictions with high and low reliabilities: "High R" and "low R" for the consistency method and "high Z" and "low Z" for the NetSurfP method, where "high" is the 50% most reliable predictions according to the chosen reliability score, and "low" is the 50% least reliable predictions.

It has previously been reported that amino acid residues, which are predicted to be highly buried tend to have lower predicted error compared to those predicted as exposed [5,22]. To investigate how this might bias the reliabilities we examined the mean predicted error as a function of the predicted exposure when splitting the data in two groups with high (top 50%) and low (bottom 50%) reliability, respectively (Figure 3). The plot visualizes how the predictions with a corresponding high Z-score have a lower mean error compared to those with a low Z-score. This is valid for all ranges of predicted exposure. This, on the other hand, is not the case for the consistency scores. Comparing the "high" and "low" reliability groups we see a difference only for residues that were predicted to be buried (RSA < 0.2). The same trend is observed when using a cut-off of top 25% and 75% highest predictions for both Real-SPINE and NetSurfP (data not shown).

**Figure 4:**
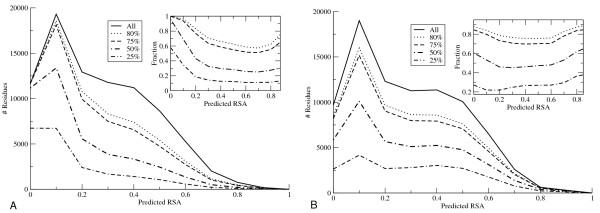
**Histogram of the number of predicted residues (A: Real-Spine and B: NetSurfP) as a function of the predicted relative exposure value for all residues in the CB511 data set at different cut-offs**. The full line shows the calculated (measured) exposure distribution of the full set. The distribution of the 25%, 50%, 75% and 80% most reliably A: Real-Spine predicted residues according to consistency score, and B: NetSurfP predicted residues according to the Z-score, are also shown. Insert shows the number of predicted residues/all predictions in a given threshold as a function of the predicted RSA.

Likewise, we tested to what degree the two reliability measures are capable of identifying reliable predictions independent of the degree of exposure. The distribution of predicted RSA values for the 25%, 50%, 75% and 80% residues with highest consistency scores was shown for the Real-SPINE (Figure 4, left panel) and highest Z-score for NetSurfP (Figure 4, right panel), respectively. These figures reveal that the Real-SPINE method predominantly assigns high consistency scores to buried residues, and when filtering out low consistency predictions mostly exposed residues are removed. This can be seen on the insert for Real-Spine (Figure 4, left panel) where there is a bias against low RSA. In contrast to this, high NetSurfP Z-score values are found for residues in all exposure ranges. The curve in the insert for NetSurfP (Figure 4, right panel), is close to horizontal meaning predictions are equally distributed over the different levels of exposure independent of Z-score reliability threshold. The predictive performance of the 80% residues with highest reliability of the two methods is 0.73 and 0.79 in terms of the PCC for the consistency and the derived Z-score methods, respectively. This difference in predictive performance is highly significant (p < 0.0001, Bootstrap exact estimate).

The above results could depend on the chosen cut-off for the fraction of most reliable predictions (80%) that were included in the test. To investigate this bias we took an increasing number of the Z-score/consistency ranked predictions and calculated the average RSA of the selected sets both regarding predicted and measured RSA. In table 3 it is shown that the predictions from the Real-SPINE with the highest consistency have a strong bias towards buried residues. Using the NetSurfP derived Z-score, no such bias was observed and the ratio between buried/exposed residues was maintained for all levels of reliability, i.e. the mean predicted relative accessibility (P-RSA) equals the mean measured (M-RSA) in each subset. In addition, the PCC of the Z-score filtered NetSurfP predictions is better within nearly all of the most reliable subsets than that of the consistency filtered Real-SPINE predictions, despite the fact that the two methods have close to identical overall performances. Furthermore, the subsets of reliable NetSurfP predictions identified by the Z-score method maintain a constant average of both the predicted surface exposure and the surface exposure calculated from experimentally solved structures independent of the degree of reliability. However, using the consistency filter on Real-SPINE predictions we saw that the average of the predicted or calculated surface exposure decreased (i.e., the relative amount of buried residues increased) as the reliability increases. The final implementation of the NetSurfP method as a web-server was done by also including the sequences (CB513 set) that were previously only used as an evaluation set. The secondary structure predictor is implemented as part of the NetSurfP web-server. The web-server is available at 

**Table 3:** Evaluation of the Real-SPINE and NetSurfP method on subsets of residues from the CB511 dataset predicted with high reliability.

		**Real-SPINE**				**NetSurfP**			
**%Top**	**N**	**RSA**	**ASA**	**P-RSA**	**M-RSA**	**RSA**	**ASA**	**P-RSA**	**M-RSA**

10	8372	0.73	0.74	0.16	0.18	0.77	0.79	0.35	0.35
20	16745	0.73	0.74	0.16	0.18	0.79	0.79	0.31	0.31
25	20931	0.73	0.74	0.17	0.19	0.79	0.79	0.30	0.30
50	41863	0.72	0.74	0.18	0.20	0.77	0.77	0.28	0.28
75	62795	0.71	0.73	0.22	0.24	0.74	0.75	0.28	0.28
80	66981	0.71	0.73	0.23	0.25	0.73	0.74	0.28	0.28
90	75354	0.70	0.73	0.25	0.27	0.72	0.73	0.28	0.28
100	83727	0.70	0.73	0.27	0.29	0.70	0.72	0.29	0.29

%Top and N give the percentage and number of residues selected. RSA and ASA give the Pearson's correlation between predicted and target for relative and absolute surface areas, respectively. P-RSA, and M-RSA give the mean predicted and mean measured RSA values, respectively, on the selected subset of residues.

## Discussion

The power of a prediction method is commonly evaluated as an overall estimate of the accuracy of the method in large-scale benchmark experiments. Such evaluation, however, provides no knowledge of the reliability of each of the individual predictions. For discrete, qualitative predictions the reliability is conventionally estimated as the difference between output scores of selected classes. For real value prediction this approach is unfeasible. Here, we have described a new reliability score method, useful for real value predictions. We have designed and implemented the method in a way that assigns reliability scores for each single real value prediction. As an example, the method has been implemented as part of a web-server to predict the relative surface accessible area of amino acids within the three dimensional structure of a protein. By nature, the reliability method is different from other procedures where reliabilities most commonly are obtained by post-processing the output [2,22]. This method was trained to assign a reliability output to each surface accessibility prediction as an inherent part of the network architecture. This output was then recomputed to a Z-score. In tests to investigate the validity of the calculated Z-score we found that the score could indeed successfully be used to filter out more reliable predictions resulting in a significantly better correlation between predicted and measured values.

The accessible surface area has been found more difficult to predict for exposed than buried amino acids and these findings are still valid [5,21,22]. However, we see that NetSurfP Z-scores enable the identification of the most reliable/unreliable predictions for both buried and exposed amino acids. This allows for identification of subsets of highly reliable predictions covering all ranges of surface exposure. This is in contrast to the consistency score, the only other surface accessibility prediction associated reliability method [22], where high reliability scores are predominantly associated with buried amino acids.

The prediction accuracy is compared to Real-SPINE 1.0 [22] as Real-SPINE 1.0 is the server that produces the consistency measures. Furthermore the newly published Real-SPINE 3.0 [23] was not available at the time of the evaluation.

## Conclusion

In the present context, the developed reliability information is especially valuable when using the surface exposed predictions to estimate other protein structure related features such as fold, B cell epitopes, phosphorylation sites, and active sites. However, the approach is generic and is potentially useful in other types of real value predictions where ANNs have been shown to produce good results.

## Methods

### Barton Evaluaon dataset, CB513/CB500

The dataset of 513 non-homologous proteins created by Cuff and Barton [25,26] consists of > 84,000 amino acids. It is commonly known as the CB513 dataset. The dataset consist of 117 sequences from the Rost and Sander dataset of 126 non-redundant proteins [27] and 396 sequences are from the CB396 dataset by Cuff and Barton [26]. No sequences in the dataset share more than 25% sequence identity. The CB513 dataset was downloaded from the Jpred section at the Barton Group's website . This dataset is solely used for final evaluations.

### Learning/Training dataset, Cull-1764

Protein sequence data was obtained from the RCSB (Research Collaboratory for Structural Bioinformatics) Protein Data Bank (PDB) [28] July 2007 using the protein culling server PISCES [29] available at . PDB was culled using the following criteria: Maximum sequence percentage identity <= 25%, Resolution <= 2.0 Å, R-factor <= 0.2, Sequence length in the range 30 – 3,000 amino acids and including full X-ray structures only. This dataset contained 2,263 PDB protein chains, but an additional 197 chains were removed due to parsing errors using the DSSP program [30] and 302 sequences were removed due to more than 25% identity to a sequence within the CB513 set. The final Cull dataset (Cull-1764) is comprised of 1,764 sequences with a total of 417,978 amino acids. Dataset named 'testset' used for optimization of parameters and procedures is always subsets/slices of the Cull-1764 dataset that have been excluded for the particular training session.

### Posion Specific Scoring Matrices

Sequence profiles as Position-Specific Scoring Matrices (PSSM) were generated for all protein chains in the Cull-1764 and CB513 dataset, using the iterative PsiBLAST program [31]. The query sequences were blasted for four iterations against a local copy of the National Center for Biotechnology Information (NCBI) non-redundant (nr) sequence database, which for speed-up purposes had been homology-reduced to less than 70% sequence identity [32]. An E-value cut-off of 1 × 10^-5 ^was used.

### Relave Solvent Accessibility

The relative solvent accessibility (RSA) is calculated as given by equation (1).

(1)

RSA is the ratio of the solvent Accessible Surface Area (ASA) of a given residue observed in the three-dimensional structure, over the maximum obtainable solvent exposed area ASA_max _for the given amino acid residue within an extended tri-peptide flanked with either glycine [4] or alanine [5] residues. Values for the accessible surface area were calculated using the DSSP program [30].

### Neural Network Training

Two types of feed-forward neural networks [33] were used in this work: the primary and secondary networks. The primary networks assign one of the classes "Buried" or "Exposed" to each amino acid (see section *Primary Neural Networks*), whereas the secondary networks predict both the real value RSA and the reliability of the prediction in form of a Z-score (see section *Secondary Neural Networks*). A gradient descent method was used to back-propagate the errors and synapses or weights were updated as previously described [34]. For the primary networks, amino acids were encoded with both PSSM values and three extra neurons for predicted Helix, Strand and Coil, thus a total of 24 neurons were used to describe an amino acid. The two-class output from the primary networks was subsequently used as input together with PSSM to the secondary neural networks. 10-fold cross-validation was used to train the networks, where 9/10 of the data was used for training and testing was performed on the remaining 1/10, named 'testset'. A graphic overview of the method is shown in Figure 1.

### Primary Neural Networks

All amino acids in the Cull-1764 dataset were divided into two discrete categories; above and below 25% RSA meaning exposed or buried amino acids, respectively. The RSA values were calculated using the extended gly-X-gly tri-peptide state as maximally exposed. In the Cull-1764 dataset the exposed and buried categories comprised 184,757 (44.2%) and 233,221 (55.8%) amino acids, respectively.

The primary neural networks were trained using window sizes of 11, 13, 15, 17 and 19, and the following number of hidden units: 10, 20, 25, 30, 40, 50, 75 and 150. This gives a total of 40 different neural network architectures for each of the 10 subsets, giving a total of 400 neural networks. The networks were trained until maximal test set performance with a maximum of 200 epochs, using a learning rate of 0.01. Final ANNs were ranked according to test set performances. Within each of the 10 training/test set groups, we added an increasing number of trained ANNs to a network ensemble from the top of the ranked list until the best test set performance was obtained.

### Secondary Neural Networks

Target values, the ratio of ASA and ASA_max_, were assigned for all examples in the Cull-1764 dataset. The ASA_max _values were calculated using amino acids in an extended ala-X-ala tri-peptide configuration. Amino acids were encoded by use of PSSM scores and two additional values for buried and exposed class predictions obtained from the primary neural networks. A 10-fold cross-validation training was done with window size of 11, and the following number of hidden units: 10, 20, 25, 30, 40, 50, 75, 150 and 200, resulting in a total of 90 neural networks. The best results were obtained using a slow learning rate of 0.005 for a maximum of 300 epochs. For each cross-validation partition, the network architecture that achieved the highest test performance was added to the final ensemble of 10 neural networks.

## Implementation of reliability predictions

To derive a method that allows for evaluation of the accuracy of each prediction, a modified feed-forward artificial neural network method was constructed. The method takes the conventional input format defined in terms of a set of input values associated with a given target value. The network produces two output values. One value is the predicted relative surface exposure, and one is a value associated with the reliability of that predicted exposure value. The error function guiding the training of the neural network is shown in Equation 2.

(2)

Here, t_i _is the target value, o_i _is the predicted exposure value, w_i _is the predicted reliability and *λ *is a parameter defining the penalty for introducing low reliability predictions. The optimal value of *λ *= 0.05 was determined in a small 5 fold cross-validation benchmark. The rational behind this error-function is that data in the training set that are marginal to the consensus motif will most likely be predicted with the highest error. If this is a systematic error, the network should be able to lower the error by learning the weight value *w*_*i *_associated with such marginal data. To avoid that all weights are assigned a value of zero, the second penalty term is introduced to balance the loss in error introduced by the weight. This term ensures that only data points that are consistently predicted with large errors are associated with weight values lower than one. The architecture is a conventional three-layer network with one input layer, one hidden layer and one output layer. The network was trained using back-propagation, and the training was stopped when the test error was minimal. Note, that the network is trained using just one target value as input, and produces two output values. Without explicit training values, the network hence learns the predicted reliability intrinsically. It does so by lowering the relative weight on data points with high error.

**Figure 5:**
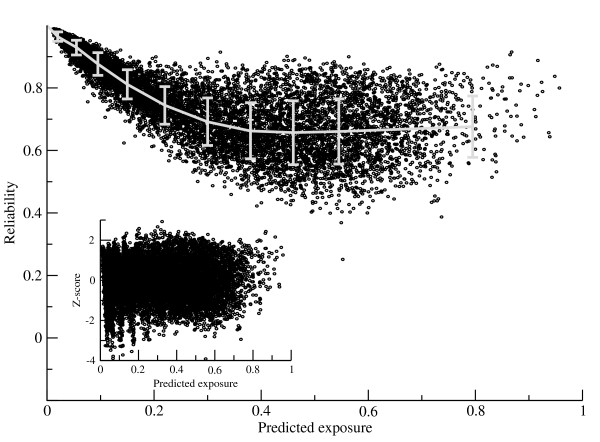
**Reliability baseline and standard deviation fitting**. The reliability is shown as a function of the predicted exposure for the Cull-1764 data set. In grey is shown the fitted reliability baseline and standard deviation. The insert shows the baseline corrected Z-scores as a function of the predicted surface exposure.

From the training it became apparent that the two output values (exposure and reliability, respectively) from the network were highly correlated. This is most likely due to the fact that deeply buried residues are relatively simple to predict and hence can be predicted with high reliability in contrast to exposed residues that have more complex characteristics. An example of this correlation is shown in Figure 5.

To allow for a direct interpretation of the predicted reliability independent of the predicted exposure value, the predicted reliability values were transformed into Z-scores using the following relation.

(3)

Here, w_0 _is the reliability baseline value at a predicted exposure value of *e*, and *σ *is the baseline-corrected standard deviation at a predicted exposure value of *e*. The reliability baseline, w_o_, and standard deviation, *σ*, were derived for each test set and network architecture from a fit to the test set predicted values. Test set predictions were grouped into 10 equally populated bins. For each bin, the baseline reliability was estimated from the prediction values in that bin. An example of the Z-score corrected reliability values is shown in Figure 5. The final Spearman's rank correlation [35] between Z-score and error is -0.19.

### Secondary Structure Predicon

Secondary structure predictions were generated for all amino acids in the dataset using an artificial neural network-based method described previously [36]. Briefly, the architecture includes combinations of primary networks predicting the three classes Helix, Extended strand or Coil with a secondary network filtering the output predictions from the primary network. For training of the method, a dataset, was downloaded from the PISCES server [29] on July 10th 2004 and consisted of 2,085 sequences with sequence identity <25%, Resolution < 2.0 Å and R-factor < 0.25. The dataset was homology-reduced with respect to the sequences in the CB513 dataset, by use of a Hobohm 1 algorithm [37]. Sequences in the CB513 dataset were used to evaluate the performance of the secondary structure predictor. Secondary structure in both sets was assigned using DSSP [30] and grouped into 3 classes: The H class comprised by DSSP class H, E class comprised by DSSP class E, and the C class comprised by the remaining DSSP classes; ., G, I, B, S and T. The method was trained using conventional 7-fold cross-validation. The final method was based on a combination of 70 primary and 70 secondary neural networks using input window sizes of 15–23 amino acids, 50 or 75 hidden units.

## Authors' contributions

BP found and curated the third party data used in this work, he performed training, evaluation and selection of ANNs for optimal surface prediction and created the first draft of manuscript. TNP have made substantial contributions to conception and design, assisted with tool and expertise for data curation and ANN development as well as revising the manuscript critically for important intellectual content. PA designed, developed, and described the secondary structure prediction algorithm. MN conceived the idea of ANN error prediction, designed the proper ANN for the task, performed the statistical analysis, and revised the manuscript critically for important intellectual content. CL initiated the development of surface accessibility prediction, and participated in its design and coordination and helped to draft the manuscript. All authors read and approved the final manuscript.
